# Cognitive Decline in Rheumatoid Arthritis: Insight into the Molecular Pathogenetic Mechanisms

**DOI:** 10.3390/ijms22031185

**Published:** 2021-01-26

**Authors:** Maria Sofia Basile, Rosella Ciurleo, Alessia Bramanti, Maria Cristina Petralia, Paolo Fagone, Ferdinando Nicoletti, Eugenio Cavalli

**Affiliations:** 1 IRCCS Centro Neurolesi “Bonino-Pulejo”, Via Provinciale Palermo, Contrada Casazza, 98124 Messina, Italy; sofiabasile@hotmail.it (M.S.B.); rossella.ciurleo@irccsme.it (R.C.); abramanti@libero.it (A.B.); m.cristinapetralia@gmail.com (M.C.P.); 2 Department of Biomedical and Biotechnological Sciences, University of Catania, Via S. Sofia 89, 95123 Catania, Italy; paolofagone@yahoo.it (P.F.); eugeniocavalli9@hotmail.it (E.C.)

**Keywords:** rheumatoid arthritis, cognitive decline, pathogenesis

## Abstract

Cognitive decline refers to a deterioration of intellectual and learning abilities and related memory problems, and is often associated with behavioral alterations, which prevents sufferers from carrying out the most common daily activities, such as maintaining normal productive interpersonal relationships, communicating, and leading an autonomous life. Numerous studies have highlighted the association between cognitive decline and autoimmune disorders, including rheumatoid arthritis (RA). RA is a chronic, inflammatory, autoimmune disease that involves systems and organs other than the bones and joints, with varying severity among patients. Here, we review the studies investigating the link between cognitive decline and RA, focusing on the main molecular pathogenetic mechanisms involved. The emerging body of data suggests that clinical, psychological, and biological factors may contribute to the pathogenesis of cognitive decline in RA, including cardiovascular complications, chronic pain, depression, inflammatory factors, changes in hormone levels, drug side effects, and genetics. Further studies are warranted in order to fully clarify the basis underlying the association between cognitive decline and RA and to find new possible diagnostic strategies and therapeutic targets for RA patients.

## 1. Rheumatoid Arthritis (RA)

RA is a chronic, inflammatory, autoimmune disease that involves systems and organs other than the bones and joints, with varying severity between patients [1]. RA is characterized by symmetrical joint pain associated with morning stiffness (joints are affected for >30 min), hyperplasia (swelling), and cartilage and bone destruction that causes rheumatoid nodules under the skin (deformity) [2,3]. However, RA not only affects the joints but is also associated with secondary amyloidosis, lymphomas, cardiovascular and pulmonary disease, vasculitis, and psychological and skeletal disorders that may cause permanent disability in many instances [2]. The presence of RA is relatively constant in the global population, with a prevalence between 0.5% and 1.0% in the European and North American populations [4]. Twice as many women are affected than men, and although it is more common in people in their fifties, it can appear at any age [4]. 

**Figure 1:**
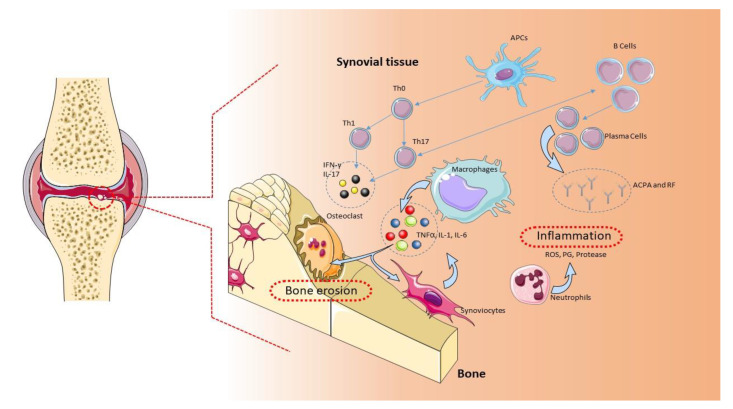
Pathogenic role of immune cells in rheumatoid arthritis (RA). The immune cells mainly involved in the pathogenesis of RA are B cells, T cells, and macrophages. These cells are normally present in the synovial tissue. B cells release proteins, such as rheumatoid factor (RF), protein antibodies (ACPAs), and proinflammatory cytokines, that support the establishment of RA. B cells also mediate the activation of T lymphocytes through the expression of costimulatory molecules. In RA, the main function of T lymphocytes is to activate macrophages. Activated T lymphocytes and macrophages release proinflammatory molecules, such as cytokines and chemokines, which keep the osteoarticular tissue inflamed. This condition favors the activation of synoviocytes and osteoclasts, with consequent damage to the osteoarticular tissue and pannus formation [1]. APCs: antigen-presenting cells, ROS: reactive oxygen species.

A complex interaction between genotype and exposure to environmental factors (cigarette smoking, air pollutants, and occupational dust) is likely to determine the onset and development of RA [5]. Genetic studies have found an association of more than 100 polymorphisms with RA, mainly in the major histocompatibility complex (MHC) locus, but also in genes encoding for cytokines/chemokines and their receptors, components of intracellular signaling pathways, and costimulatory factors [6]. The implication of genetic factors in the RA pathogenesis is demonstrated by a positive family history, which increases the risk of RA by about three to five times [4,7]. Along the same lines, a concordance rate of 15% to 30% is observed among monozygotic twins, while only a 5% concordance can be observed among dizygotic twins [1]. The characteristic chronic inflammation of the joints suggests an autoimmune origin of the pathology [8]. Typical histological findings are the symmetrical synovial proliferation, with the destruction of cartilage and bone damage induced by the activation of self-reactive T and B lymphocytes, which produce proinflammatory cytokines and autoantibodies [9] (Figure 1). Evidence obtained from preclinical and clinical studies from animal models of RA and RA patients demonstrates that CD4+ T cells belonging to the proinflammatory subgroups Th1 and Th17, together with M1 macrophages, contribute to the development and maintenance of RA and counteract the action of Th2 and Th3 anti-inflammatory cells and M2 macrophages [8].

## 2. Diagnosis and Treatment of RA

RA is a symmetrical polyarthritis with a gradual and persistent chronic course that primarily involves the joints of the hands and feet [4]. The key features of inflammatory arthritis are the presence of early morning stiffness, joint swelling affecting more than three joints, and tenderness across the metacarpo- and metatarso-phalangeal joints, as evaluated by the “squeeze test.” Axial joint involvement is less common, with cervical spine involvement occurring in 30–50% of cases, but rarely in isolation. Temporo-mandibular and crico-arytenoid joints may also be affected [10]. However, the clinical presentation and the course of the disease varies among patients, with some having very acute onset polyarthritis [4].

The European League Against Rheumatism (EULAR) Disease Activity Score (DAS-28) includes the evaluation of the number of affected joints, the level of acute inflammatory markers, and the patient global well-being score [10]. RA is characterized by the presence of rheumatoid factor (RF) and anti-citrullinated protein antibodies (ACPAs). Positive serology for RF can be found in 60–80% of patients with established RA, but it is less frequently present in early RA (<50%). ACPAs have a specificity of 94–97% and a sensitivity of 62–72% in early RA. ACPAs can be detected early in the course of RA, often appearing before RF, and may be observed before clinical manifestations of the disease. In ACPAs-negative patients, other autoantibodies have been observed, including anti-carbamylated proteins [11], anti-malondialdehyde acetaldehyde [12], anti BRAF [13], 14-3-3eta, anti-CarP, anti-Sa [14], and anti PAD3/PAD4 [15] antibodies.

Non-steroidal anti-inflammatory drugs (NSAIDs), such as ibuprofen, naproxen, ketoprofen, piroxicam, diclofenac, and Celecoxib, are widely used as symptomatic therapies [16]. Disease-modifying antirheumatic drugs (DMARDs) are a heterogeneous collection of agents that represent the mainstay of treatment for RA [16]. DMARDs can be classified as conventional synthetic DMARDs (csDMARDs, which include methotrexate, sulfasalazine, leflunomide, and hydroxychloroquine), targeted synthetic DMARDs (tsDMARDs, which include tofacitinib and baricitinib), and biological DMARDs (bDMARDs, such as adalimumab, tocilizumab, secukinumab, abatacept, and anakinra). Methotrexate represents the first choice csDMARD. Leflunomide or sulfasalazine is used in the case of contraindication to methotrexate. In patients not responding to treatment, or in the presence of poor prognostic factors, it is recommended to add a bDMARD or tsDMARD. Steroids, although effective in reducing pain and disease progression, are used temporarily as an adjunctive treatment because of their side effects [17].

## 3. Cognitive Decline

Cognitive decline can be defined as a psychophysical condition that is characterized by an alteration in the orientation, attention, problem-solving abilities, memory, and executive functions [18]. Cognitive impairment may range from a subtle decline in a single cognitive domain to impairment in multiple cognitive domains (mild cognitive impairment (MCI)) to frank dementia, which is characterized by cognitive decline and the loss of function. To diagnose MCI, the following parameters should be met: complaint of a decline in cognitive function, impairment of one or more cognitive domains, and independent function preserved, with no alteration in social and work skills [19]. 

Impaired episodic memory is typically seen in patients with MCI, who may later progress to dementia. Alzheimer’s disease (AD) is the most common form of dementia and accounts for 50–70% of dementia cases [20].

The growth of the aging population has been associated with an increased burden from cognitive disorders. The prevalence of MCI ranges from 12% to 18% among the older adults (≥65 years old) with an annual 10–15% conversion rate to AD [20]. 

Cognitive impairment can arise from many chronic diseases, such as hypertension, dyslipidemia, vascular disease, diabetes, chronic obstructive lung disease, depression, anxiety, autoimmune diseases, epilepsy, and drug dependency. Head injuries can lead to impaired cognition. Furthermore, anti-depressants, anticonvulsants, and antipsychotics are associated with cognitive decline. However, in most of these conditions, cognitive disorders are treatable, particularly when they are detected early through monitoring. 

For the diagnosis of cognitive decline, different standardized tests have been developed, including the Mini-Mental State Examination (MMSE), the Montreal Cognitive Assessment (MoCA), the Trail-Making Test (TMT), the Victoria Stroop Test (VST), the Wechsler Adult Intelligence Scale (WAIS), and the Benton Visual Retention Test (BVRT) [21]. The Beck Depression Inventory (BDI) and the State-Trait Anxiety Inventory (STAIT/S) are used to assess the presence of depression and anxiety, which are commonly found in RA patients [21].

## 4. RA and Cognitive Decline

An increased risk of cognitive decline has recently been associated with the presence of rheumatic diseases [22,23]. It is hypothesized that the triggering cause could be represented by the systemic inflammation that is associated with a chronic rheumatological condition [22,23]. In particular, numerous studies have shown the presence of a cognitive decline in RA patients [18].

**Figure 2:**
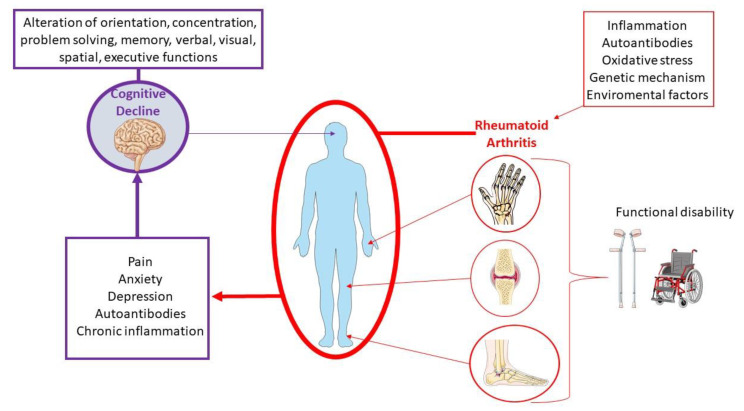
Multistep progression of rheumatoid arthritis to cognitive dysfunction.

To date, the molecular pathogenetic mechanisms that underlie the association of cognitive decline and RA are not fully clarified. However, during the last few years, a growing number of studies have investigated the link between these conditions, highlighting the potential pathogenic role of several clinical, psychological, and biological factors (Figure 2). These include cardiovascular complications and chronic pain, along with the involvement of autoimmune and inflammatory factors, changes in hormone levels, drug side effects, genetic factors, and psychiatric disorders [18,22,24,25,26].

Among the psychiatric conditions, depression and anxiety are mostly associated with RA patients [27]. Usually, the peak of onset of the disease occurs in individuals during their professional and social life, thus compromising the social sphere [28,29]. It has been observed that depression affects up to 66% of patients, anxiety affects 70% of patients, and nearly 17% of RA patients have a major depressive disorder [30,31,32,33]. Depression is usually associated with higher levels of pain and disability, resulting in a lower health-related quality of life and increased mortality [34,35]. Moreover, RA disease activity has been associated with cognitive decline [36,37]. 

Several studies have reported that the association between cognitive decline and RA is more evident in patients of advanced age [38,39,40,41]. A debilitating condition leading to cognitive decline is associated with the recorded increase in accelerated inflammatory atherosclerosis with a consequent risk of stroke, especially in elderly RA patients with long-standing illness [42]. However, cognitive decline has also been observed in young RA patients, in particular during the early stages of the disease [43].

Other risk factors for cognitive decline in RA include cardiovascular risk and the use of certain drugs, such as glucocorticoids [38]. However, there are some studies that found no association between cardiovascular risk or medication intake and cognitive decline in RA patients [36,44]. On the other hand, treatment with biological anti-tumor necrosis factor (TNF) therapies in RA patients may be protective, showing a lower risk percentage of developing cognitive decline [39].

In this review, we analyzed and explored the correlation between cognitive decline and RA from a pathogenic point of view, focusing on the main molecular mechanisms involved.

## 5. Clinical and Psychological Factors

Among the clinical and psychological factors that may be involved, the presence of cardiovascular complications, chronic pain, and depression seems to be particularly relevant in the onset and development of cognitive decline in RA patients.

### 5.1. Cardiovascular Complications

It is known that RA patients have a higher risk of cardiovascular diseases (e.g., stroke and myocardial infarctions) [39]. The common molecular mechanisms that relate RA and cardiovascular diseases are inflammatory mediators, post-translational modifications of peptides and/or proteins and the consequent immunological response, changes in lipoprotein levels, higher oxidative stress, and endothelial dysfunction [45]. Interestingly, it has been suggested that the increased risk of cardiovascular diseases in RA patients could be involved in cognitive decline via mechanisms associated with metabolic syndrome and inflammatory proteins [18]. On the other hand, it is known that the risk of dementia is higher in the presence of cerebrovascular dysfunctions and that cerebral small vessel disorder may impair cognitive and cerebral functions, thus suggesting other possible links between dementia and RA [39]. However, it has also been shown that there is no correlation between cognitive function and carotid atherosclerotic changes in patients with RA, thus suggesting the need for more studies in this field [36]. 

### 5.2. Chronic Pain

Previous studies have shown that cognitive functions may be affected by pain in RA patients [46]. Although pain may be considered a useful signal of present or possible injury, chronic pain could damage attention and memory and might impair the capability to work, sleep, and carry out daily life activities, which usually worsens over time [46]. Even though it is known that chronic pain negatively influences cognitive functions, such as memory, attention, and mental flexibility, the exact mechanism underlying pain-related cognitive impairment should be clarified [47]. In particular, it has been suggested that two possible factors could be involved. On the one hand, this might be due to some overlaps between the brain regions involved in cognition and pain modulation, such as the prefrontal cortex and the anterior cingulate cortex [39,47]. On the other hand, this could largely derive from hypervigilance to pain [39,47]. Indeed, patients focus their attention on coping with pain, thus decreasing their cognitive task performance [47].

### 5.3. Depression

RA patients also have a higher prevalence of mood disorders than the general population and, in particular, depression is the psychiatric condition most frequently associated with RA [21,48]. Of note, cognitive decline could arise either along with depression or independently from this factor [21]. It is known that depression may cause problems with concentration and executive functions and that cognitive decline could be a feature of depression [18]. The close relationship between peripheral and brain immune responses suggests that immune-mediated inflammatory disorders and depression, which often coexist, could share different pathophysiological mechanisms [49]. Indeed, it has been proposed that proinflammatory mediators may negatively impact monoaminergic neurotransmission and the maintenance of synaptic plasticity [49]. In addition, as we reviewed elsewhere, increasing evidence indicates that the augmented levels of proinflammatory cytokines, such as interleukin (IL)-6, IL-1β, TNF-α, and macrophage migration inhibitory factors that are found in RA patients, may play a role in the induction of depression through multiple biological mechanisms [49,50,51,52]. That proinflammatory cytokines may be implicated in the pathogenesis of depression concurs with the observation that other immunoinflammatory and autoimmune diseases associated with upregulated production of these cytokines, such as psoriasis and multiple sclerosis, are associated with depression [53,54,55,56].

Note, however, that the polarization toward the upregulated production of proinflammatory cytokines may not entirely account for the induction of depression, as autoimmune diseases characterized by combined upregulated production of type 1 (proinflammatory) and type 2 (anti-inflammatory) cytokines, such as systemic lupus erythematosus (SLE) [57], are also associated with depression. In particular, in SLE patients, depression has been shown to correlate with the levels of the anti-inflammatory cytokine IL-10 [58].

Hence, it is likely that the abnormal functioning of the immune system may dictate the outcome of depression acting in concert with different genetic, environmental, and pharmacological factors. Nonetheless, and of particular relevance for the topic of this review, not only there is a consensus that the augmented production of proinflammatory cytokines plays a role in the pathogenesis of depression in RA but it is also emerging that their augmented levels may be used as a personalized parameter to predict the risk of depression and aid the design of tailored therapeutic approaches. Along this line, it is of interest that RA patients that received TNF-α inhibitors as a standard-of-care treatment for RA also exhibited an improvement regarding their depression [59].

These observations could not only contribute to clarifying the increased prevalence of depression among RA patients but could also shed new light on the possible biological mechanisms involved in the link between depression, cognitive decline, and RA.

## 6. Biological Factors

The cognitive dysfunctions that are associated with RA are often regarded as a consequence of functional disabilities, drug-related effects, and chronic pain. This is supported by electroencephalography and brain MRI data that show increased responses to nociception in RA patients as compared to healthy people [60,61]. However, although no global differences in intracranial volume were observed between RA patients and healthy subjects, a significant increase in the volume of basal ganglia was documented [62]. In particular, Wartolowska et al. found that the caudate nucleus had the most significant increase, which led to the conclusion that these structural changes may be due to pain processing rather than a direct consequence of the disease. Despite this data, other studies have shown both augmented and reduced gray matter structure volumes in patients suffering from chronic pain, suggesting that several factors could be involved, such as neurodegeneration, adaptive changes to the disease, pharmacological treatment, and changes in lifestyle [62]. Bekkelund and colleagues observed that only RA patients with a long-established disease (>15 years) have a decrease in brain volume, which could be related to neurodegenerative changes [63].

**Figure 3:**
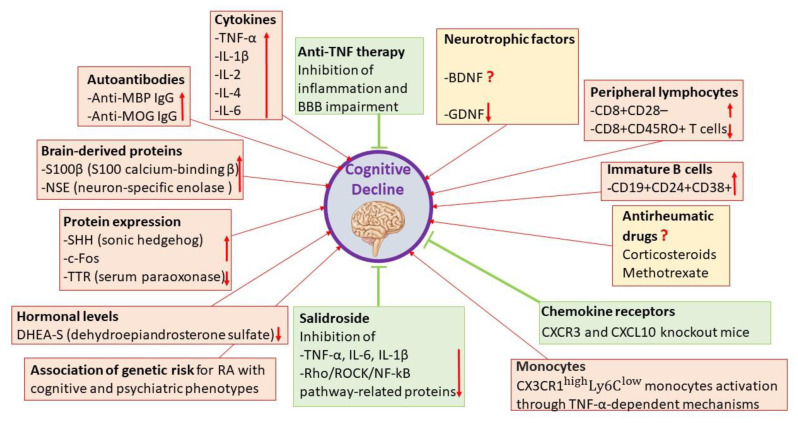
Overview of the possible biological factors involved in the pathogenesis of cognitive decline in rheumatoid arthritis.

Regarding the biological factors, autoimmune and inflammatory factors, along with changes in hormone levels, drug side effects and genetic factors seem to represent the links between cognitive decline and RA (Figure 3).

### 6.1. Autoimmune and Inflammatory Factors

#### 6.1.1. Premature Immunosenescence

Petersen and colleagues studied the link between cognitive functions and peripheral lymphocyte subsets in RA patients [64]. In comparison to controls, patients showed an expansion of CD8+CD28− cells and a reduction of memory CD8+CD45RO+ T cells [64]. Of note, CD8+CD28− and CD8+CD45RO+ T cells were associated with memory decline [64]. In light of these results, accelerated immunosenescence could be involved in the relationship between memory dysfunction and RA [64].

#### 6.1.2. Autoantibodies and Brain-Derived Proteins

Baptista et al. investigated the circulating levels of autoantibodies in order to evaluate whether there was a link between these parameters and cognitive functions in RA patients with active disease [25]. Interestingly, they found that cognitive performance was negatively associated with the levels of the anti-myelin basic protein (MBP) IgG, the anti-myelin oligodendrocyte glycoprotein (MOG) IgG, and S100 calcium-binding β (S100β) [25]. The authors hypothesized that the augmented permeability of the blood–brain barrier (BBB) might represent the trigger of the pathophysiological mechanisms of cognitive decline in RA [25]. Indeed, antibodies and inflammatory mediators might be able to reach the cerebral parenchyma, leading to an exaggerated release of neurotoxic factors, thus promoting neuroinflammation, along with demyelination processes that are induced by anti-MOG and anti-MBP antibodies [25]. Overall, these factors could negatively alter the number of neurons and synapses, as well as information processing speed and neurogenesis, leading to cognitive decline [25]. 

Considering that the central nervous system’s involvement in RA might derive from BBB damage associated with chronic inflammation, Sag et al. examined the potential role of BBB damage and evaluated the action of TNF blocker therapy on BBB function in RA patients [65]. They found that S100β and glial fibrillary acidic protein (GFAP) levels were significantly increased in patients compared to controls [65]. Interestingly, the group treated with TNF blocker therapy showed significantly reduced levels of S100β and GFAP after 6 months from the start of the treatment [65]. The S100β levels increased in RA patients, along with lesions in the deep white matter examined with cranial magnetic resonance imaging (MRI) [65]. Overall, the authors suggested that the anti-TNF therapy in RA could both reduce disease activity and joint erosions by inhibiting inflammation and block the possible involvement of the central nervous system in BBB impairment [65]. 

Furthermore, Hamed et al. evaluated the serum levels of S100β and neuron-specific enolase (NSE) in female RA patients [66]. Of note, they found that, compared to controls, patients showed increased concentrations of S100β and that increased concentrations of S100β were associated with worse cognitive performance and increased concentrations of NSE [66]. These data are in line with previous studies suggesting that increased levels of NSE may enhance neuroinflammatory processes, oxidative stress, and neuronal apoptosis [66]. These results highlight the potential diagnostic importance of the assessment of the serum levels of certain specific brain-derived proteins, such as S100β and NSE, in RA patients in order to identify the cognitive dysfunction that is associated with the brain injury subsequent to inflammation [66]. 

#### 6.1.3. Proinflammatory Cytokines

Chronic inflammation with high circulating levels of proinflammatory cytokines and sustained brain cytokine production is considered to be the leading cause of cognitive impairment [67,68]. In addition to cytokines, autoantibodies, including the RF [69,70] and immune complexes, can also induce neuroinflammatory responses in the brain [71]. Indeed, in a murine model, it was shown that immune complexes in the brain parenchyma elicited inflammation, along with augmented microglial expressions of CD11b, CD68, and FcRII/III, as well as consequent neuronal damage, in the absence of neutrophil recruitment [71]. Importantly, these effects were dependent on the Fcγ receptors but not on the complement system [71].

In the cerebrospinal fluid (CSF) of RA patients, IL-1β levels have been found to be significantly increased when compared with controls. Interestingly, CSF IL-1β levels were higher in comparison to the serum levels, indicating that RA is associated with brain immune activation, despite the lack of other markers of systemic inflammation. Concordantly, Lampa et al. observed a decrease in IL-1R antagonist (IL1Ra) in RA CSF. On the other hand, no significant differences were found in CSF TNF-α levels between RA patients and healthy controls, while a trend toward an increase was observed for IL-6 [72]. 

It has been suggested that cytokines, such as IL-1β and TNF-α, can modulate the excitability of neurons, not only via interaction with their receptors but also via noncanonical signaling pathways. In particular, IL-1β and TNF-α are able to modulate the main types of voltage- and ligand-dependent membrane channels in brain cells (reviewed by [73]). At the cellular level, IL-1β is able to inhibit the activity of glucose-sensitive neurons of the lateral hypothalamus to promote the production of vasopressin in the hypothalamus, to diminish the GABA-mediated inhibition of Purkinje cells in the cerebellum, to hinder the glutamatergic transmission in the hippocampus, and to inhibit the N-type voltage-gated Ca^2+^ channels. Moreover, IL-1β favors astrocyte and microglial proliferation, stimulates angiogenesis in the brain, and increases blood vessel permeability (reviewed by [73]). On the other hand, TNF-α is able to increase the expression of AMPA glutamate receptors and to reduce the expression of GABA-A receptors in the hippocampus, consequently controlling the plastic changes in the neural networks of this region (reviewed by [73]). Moreover, TNF-α can enhance the outward K^+^ current in cortical neurons and reduce glutamate-induced currents in hippocampal neurons via the NF-κB pathway (reviewed by [73]). 

These data support the notion that cytokines have a significant role in the modulation of synaptic plasticity and could regulate memory formation and cognitive function. However, the effect of TNF-α in learning and memory could be age-dependent. Indeed, older mice chronically overexpressing neuronal TNF-α display spatial memory impairments, while no such deficits are observed in young (30-day-old) mice [74,75].

A negative role for IL-1β regarding learning and memory has been demonstrated in rodent models of chronic elevated IL-1β levels in the brain. The chronic injection of IL-1β into the lateral ventricles has been reported to induce spatial memory deficits in rats [76]. Moreover, chronic hippocampal overexpression of IL-1β in mice caused an increase in glial inflammatory markers, increased the production of cytokines and chemokines in the hippocampus, and lowered the levels of the *Arc* gene, which is associated with neuron plasticity [77,78]. Accordingly, these mice showed decreased retention of spatial memory and fear memory [77,78].

Furthermore, a detrimental effect of IL-6 on cognition has been described [79]. As compared to wild-type (WT) mice, IL-6 knockout (KO) animals did not display working memory impairment and lacked the expected LPS-induced increase in TNF-α and IL-1β in the hippocampus [80], suggesting that IL-6 is required for the LPS-induced production of TNF-α and IL-1β in the brain and the development of behavioral impairments. Furthermore, mice chronically expressing astrocytic IL-6 display a progressive age-related decline in learning performance that correlates with presynaptic loss [81] and a decrease in cortical and hippocampal neuronal calbindin [82].

Interestingly, Chou et al. found that RA patients receiving an anti-TNF treatment (infliximab, etanercept, and adalimumab) had a reduced risk of developing AD as compared to controls [83]. Contrarily, the risk of developing AD was not changed by treatment with other DMARDs. More importantly, the impact of anti-TNF treatment on cognition seems to occur before its anti-inflammatory effects become clinically apparent in the joints. In a functional MRI (fMRI) study [84], upon anti-TNF-α therapy in patients with active RA, brain activation was significantly decreased within 3 days after treatment, while the disease activity score was significantly reduced only by day 28. Hence, the improvement in cognitive decline observed upon anti-TNF-α therapy may be a direct central effect and may not be subsequent to the better management of pain in RA patients.

#### 6.1.4. Lymphocyte Subsets, Chemokines, and Neurotrophic Factors

Petersen et al. evaluated the cognitive functions of RA patients with controlled and active disease and investigated whether cognitive decline was associated with immune and neurotrophic markers, such as lymphocyte subsets, cytokines, and neurotrophic factors, in RA patients [85]. They found an overall cognitive impairment in RA patients and the cognitive performance was worse in RA patients with active disease than in those with controlled disease [85]. Moreover, in comparison to controls, RA patients showed an expansion of some lymphocyte subsets, such as natural killer T cell (NKT) cells and CD4+IL-17+ T cells, as well as a reduction of regulatory T cells [85]. Furthermore, RA patients were characterized by an expansion of immature B cells (CD19+CD24+CD38+) and plasma cells (CD19+CD27+CD38+) compared to controls, and low cognitive scores were correlated with bigger proportions of immature B cells [85]. Furthermore, RA patients revealed higher TNF-α, interleukin IL-2, IL-4, and IL-6 plasma levels, which was negatively correlated with cognitive functions [85]. 

Moreover, chemokines and the complement system have been found to be important mediators that are involved in CNS homeostasis [86,87]. Garré et al. showed significant impairments in dendritic spine formation and in learning abilities upon treatment with polyinosinic:polycytidilic acid (poly I:C), and that activation of CX3CR1^high^Ly6C^low^ monocytes impaired motor learning and learning-related dendritic spine plasticity via TNF-α-dependent mechanisms [88]. Moreover, Blank et al. showed that the production of CXCL10 by brain endothelial and epithelial cells was associated with diminished hippocampal plasticity, and that CXCR3^−/−^ and CXCL10^−/−^ mice retained better memory and learning functions [89], supporting the notion that inflammatory factors could have a role in cognitive decline.

Moreover, increased brain-derived neurotrophic factor (BDNF) levels and decreased glial-cell-line-derived neurotrophic factor (GDNF) levels were found in RA patients when compared to controls [85]. 

Overall, these data demonstrated a global cognitive decline in RA patients, which was associated with disease activity and immune differences, thus suggesting that peripheral immune imbalance, along with a proinflammatory milieu, could predict the cognitive deficits in RA. Of note, although the presence of higher BDNF plasma levels could seem unusual in patients with chronic inflammation, such as those with RA, it should be considered that the circulating BDNF might largely be derived from leukocytes in inflammatory diseases [90]. Interestingly, it has been shown that BDNF is constitutively expressed by peripheral blood mononuclear cells (PBMCs) and synovial cells [90]. Instead, GDNF, whose decreased plasma levels were found to be associated with cognitive dysfunctions in RA patients, is produced only in the central nervous system [90]. Therefore, decreased levels of GDNF could be a better predictor of worse cognitive performance compared to BDNF [90].

On the other hand, Pedard et al. explored the role of the cerebral BDNF pathway in a preclinical rat model of RA [91]. This study demonstrated that arthritis was negatively associated with the cerebral BDNF/tropomyosin-related kinase B (TrkB) pathway both at the endothelial and neuronal levels, without correlation with the severity of inflammatory symptoms, but they were dependent on endothelial nitric oxide (NO) production [91]. In particular, it is suggested that reduced BDNF production by the cerebral endothelium, deriving from reduced endothelial NO synthesis, could explain the arthritis-associated reduced activation of neuronal TrkB activation [91]. These results might shed new light on clarifying the relationship between cognitive and endothelial dysfunctions, which are both present in RA [91].

#### 6.1.5. Differential Protein Expression

Yang et al. investigated the differentially expressed proteins that might be possible biomarkers for a differential diagnosis of MCI in RA patients [26]. The authors compared plasma protein levels from RA patients with and without MCI and from healthy controls [26]. Interestingly, 14 differentially expressed proteins, 6 upregulated and 8 downregulated, were identified in RA patients with MCI [26]. These dysregulated proteins are implicated in numerous biological processes and pathways, such as immunity, inflammation, and coagulation [26]. In particular, sonic hedgehog (SHH) and serum paraoxonase (TTR), which were respectively upregulated and downregulated in RA patients with MCI, seem to be promising potential plasma biomarkers for the diagnosis of MCI in RA patients [26].

Carter et al. investigated the levels of c-Fos expression in the hippocampus, which is a brain region that is crucially involved in cognitive function, in a preclinical rodent model of RA, namely, the adjuvant-induced arthritis Lewis rat model [92]. A persistent dose- and subfield-dependent expression of c-Fos was found in the arthritis group, whereas a transient expression was found in groups without arthritis [92]. The mechanisms that cause c-Fos expression in the hippocampus were not identified in this study [92]. Nonetheless, other studies have previously shown that immunization with different immunogens, such as lipopolysaccharide or proinflammatory cytokines, including TNF-α or IL-1, may induce a sustained c-Fos immunoreactivity in the hypothalamic, limbic, and autonomic brain areas [92]. 

The reason for the persistent increase in c-Fos expression in the hippocampus of adjuvant-induced arthritis rats is not yet fully understood [92]. It is known that c-Fos accumulates when its C-terminus is phosphorylated in the presence of sustained ERK activation, thus indicating that hippocampal pyramidal cells in adjuvant-induced arthritis rats may show a chronic increase in ERK activity [92]. Moreover, the higher stability of c-Fos by phosphorylation increases ERK phosphorylation at its C-terminus [92].

Changes in c-Fos subsequent to sustained ERK signaling in adjuvant-induced arthritis might chronically modify pyramidal cell functional processes; thus, this might favor disease progression and could modify behavior and cognitive functions in adjuvant-induced arthritis [92].

Overall, these results suggest that the chronic expression of c-Fos in the hippocampus of the adjuvant-induced arthritis rats might influence several cell functions, such as synaptogenesis, electrical activity, and neurotransmitters, and that sustained genomic alterations in RA could be involved in different processes associated with RA, including cognitive decline [92].

#### 6.1.6. Rho/ROCK/NF-κB Pathway

Considering the increasing prevalence of cognitive impairment in RA patients and the increasing lines of evidence about the role of inflammation in arthritis-induced cognitive deficits, Zhu et al. investigated the effects of Salidroside (Sal, p-hydroxyphenethyl-b-D-glucoside), which is an effective extracted ingredient of *Rhodiola rosea* L with anti-inflammatory properties, on the arthritis-induced cognitive dysfunction in a preclinical rat model of collagen-induced arthritis [93]. The results of this study showed that Sal exerted a protective action on arthritis-induced cognitive dysfunction through the inhibition of proinflammatory cytokines (TNF-α, IL-6, and IL-1β) and the regulation of the Rho/ROCK/NF-κB pathway [93]. Systemic proinflammatory cytokines may pass through the damaged BBB and reach the central nervous system, thus stimulating neurodegenerative processes [93]. Moreover, according to previous studies, the Rho/ROCK/NF-κB pathway may be implicated in cognitive impairment [93]. The Rho/ROCK pathway is usually involved in the production of proinflammatory factors [93]. Even though the exact cellular processes of Rho signaling in the central nervous system have yet to be clarified, it is known that changes in Rho signaling derived from mutations cause anomalous neuronal connectivity and cognitive deficits in humans [93]. Furthermore, small G proteins of the Rho family are involved in different biological processes and in the regulation of several signaling pathways that are correlated with inflammation, including the NF-κB pathway [93]. Of note, Rho proteins may regulate cell adhesion via transmembrane proteins, such as cadherins and integrins [93]. In particular, cadherins are involved in synaptic plasticity, thus suggesting that Rho proteins could be implicated in the regulation of synapse formation and plasticity [93]. It is known that synapses provide the structural basis that regulates higher brain functions, including learning and memory, and that damaged synaptic plasticity and neurotransmission due to inflammation may alter cognitive functions [93]. 

Overall, these results highlight the role of inflammation in affecting brain functions and suggest the involvement of the Rho/ROCK/NF-κB signaling and its potential as a possible novel molecular target [93].

### 6.2. Changes in Hormonal Levels

Kozora et al. found that RA patients with mild levels of disease activity showed significantly decreased plasma levels of dehydroepiandrosterone sulfate (DHEA-S) compared to controls and that reduced plasma levels of DHEA-S were marginally associated with reduced scores on measures of attention [94]. Interestingly, previous studies have shown an association between dehydroepiandrosterone (DHEA) and DHEA-S and cognitive functions, both in animal models and in humans [94]. Moreover, it is known that metabolites of DHEA may play an important role in immune regulation [94]. However, further studies are needed in order to clarify the mediators of these cognitive differences and to further confirm these data [94]. Overall, the results of this study shed light on the potential value of hormones as predictors of cognitive function in RA patients [94].

### 6.3. Drug Side Effects 

It has been shown that some drugs that are commonly used to treat RA, including methotrexate and corticosteroids, could be associated with cognitive dysfunction in RA [18,22]. However, the question seems to be controversial and the possible mechanisms involved are not clear. Indeed, it is known that the anti-inflammatory action of methotrexate and corticosteroids may exert positive effects on cognitive functions [22]. Nonetheless, methotrexate could be associated with cognitive decline, confusion, and mood changes, whereas corticosteroids could influence memory and hippocampal function [18,22]. Moreover, cognitive decline has been associated with current or long-term steroid use in RA patients, probably due to its vascular side effects [24]. On the other hand, other studies have shown that there is no significant correlation between cognitive decline and glucocorticoid use for RA [44,90].

### 6.4. Genetic Factors

Jones et al. explored whether genetics could be related to cognitive and psychiatric phenotypes in children and adolescents before the clinical onset of RA [95]. The authors identified a polygenic risk score for RA, which was associated with decreased scores on some measures of cognition, such as total IQ, performance IQ, and verbal IQ, along with significantly higher associations with hyperactive and inattentive symptoms [95]. Moreover, trends toward negative associations with working memory (*p* = 0.058) and verbal learning (*p* = 0.174) were also found. The results of this study highlight the presence of a relationship between genetic risk for RA and neural phenotypes, thus indicating that cognitive decline in RA is not just a result of disease-related processes or drug effects, but it depends on more complex interactions that also involve genetic susceptibility and immune factors [95].

## 7. Conclusions

To date, numerous studies have highlighted the association between cognitive decline and RA and have investigated the underlying potential mechanisms. In this review, we examined the link between cognitive decline and RA from a pathogenic point of view, focusing on the main molecular mechanisms involved. Although the molecular pathogenetic mechanisms underlying the association of these conditions are not still fully clarified, the emerging results from the preclinical and clinical studies in this field suggest that different clinical, psychological, and biological variables may contribute to the pathogenesis of cognitive decline in RA. Regarding the clinical and psychological variables involved, the presence of cardiovascular complications, chronic pain, and depression seem to be particularly relevant. Among the biological variables, several autoimmune and inflammatory factors, along with changes in hormone levels, drug side effects, and genetic risk, appear to be involved. Overall, inflammation seems to be the main actor in this scenario. Interestingly, premature immunosenescence, autoantibodies, and brain-derived proteins, as well as alterations in signaling pathways, lymphocyte subsets, cytokines, and neurotrophic factors, might be contributing mechanisms. 

Regarding the potential diagnostic and prognostic strategies and the identification of molecular targets for cognitive decline in RA, the emerging results from the reviewed studies suggest different possibilities. These include the potential diagnostic importance of the assessment of the serum levels of certain specific brain-derived proteins, such as S100β and NSE, in order to identify the cognitive dysfunction that is associated with brain injury [66]; the decreased levels of GDNF as a predictor of worse cognitive performance [90]; the evaluation of SHH and TTR as potential plasma biomarkers for the diagnosis of MCI [26]; the Rho/ROCK/NF-κB signaling as a novel molecular target [93]; the determination of hormones as predictors of cognitive function [94].

Since inflammation, which characterizes RA, could be considered the most important molecular pathogenetic mechanism involved in the cognitive decline associated with RA, the use of different anti-inflammatory drugs in RA patients might acquire added value. Indeed, the anti-inflammatory drugs, most of which are commonly used in RA treatment, could exert a therapeutic action not only on the progression of RA but also on the development of cognitive decline in RA patients. Therefore, the possibility to evaluate novel pharmacological classes in order to select the most effective ones, and eventually finding out new possible synergistic co-treatments, could be evaluated. However, it should be noted that certain drugs, such as methotrexate and corticosteroids, might have a controversial role in this field. Indeed, despite their anti-inflammatory action, which may exert positive effects on cognitive functions, certain studies have suggested that these drugs might be associated with cognitive dysfunction in RA [18,22]. However, other studies on this topic, in particular on glucocorticoids, have found no correlation [44,90].

Among the anti-inflammatory therapies, the anti-TNF-α therapies seem to be particularly promising. Interestingly, it has recently been shown that RA patients treated with TNF-α-inhibiting biological therapies showed a 50% decreased risk of developing cognitive decline, where this may be due to the fact that TNF-α is involved in the physiopathology of dementia, as well as in that of RA [39].

Along this line of research, a recent clinical trial (NCT04378621) aimed to examine how RA influences the brain structures in RA patients and whether anti-inflammatory treatments targeting TNF-α or JAK signaling, as compared to the physical training of hands, exert a positive effect on neuropsychiatric symptoms, including cognitive decline, and on morphological changes in the brain derived from the disease. The TNF-α inhibitors considered for this study were Etanercept, Infliximab, Adalimumab, Certolizumab pegol, and Golimumab, whereas the JAK inhibitors were Baricitinib and Tofacitinib. 

Understanding the molecular basis underlying the link between cognitive decline and RA is of fundamental importance to find out new possible diagnostic, prognostic, and therapeutic strategies; this can be done by focusing on the discovery of novel potential biomarkers, therapeutic targets, and treatments for RA patients. Of note, targeting immune pathways could be a potentially valuable therapeutic approach. Considering the strong interaction between mental and physical dysfunctions, a multidisciplinary approach that aims to target all the variables involved seems to be promising. Further studies are highly warranted in order to fully clarify the association between cognitive decline and RA.

## Abbreviations

ACPAs	anti-citrullinated peptide antibodies
AD	Alzheimer’s disease
BBB	blood–brain barrier
BDI	Beck Depression Inventory
bDMARDs	biological DMARDs
BDNF	brain-derived neurotrophic factor
BVRT	Benton Visual Retention Test
csDMARDs	conventional synthetic DMARDs
CSF	cerebrospinal fluid
DAS-28	Disease Activity Score
DHEADHEA-S	dehydroepiandrosterone dehydroepiandrosterone sulfate
EULAR	European League Against Rheumatism
fMRI	functional MRI
GDNF	glial-cell-line-derived neurotrophic factor
GFAP	glial fibrillary acidic protein
MBP	myelin basic protein
MCI	mild cognitive impairment
MHC	major histocompatibility complex
MMSE	Mini-Mental State Examination
MoCA	Montreal Cognitive Assessment
MOG	myelin oligodendrocyte glycoprotein
MRI	magnetic resonance imaging
NKT	natural killer T cell
NO	nitric oxide
NSAIDs	non-steroidal anti-inflammatory drugs
PBMCs	peripheral blood mononuclear cells
poly I:C	polyinosinic:polycytidilic acid
RA	rheumatoid arthritis
RF	rheumatoid factor
S100β	S100 calcium-binding β
Sal	salidroside
SHH	sonic hedgehog
SLE	systemic lupus erythematosus
STAIT/S TNF	State-Trait Anxiety Inventory Tumor necrosis factor
TMT	Trail-Making Test
TrkB	tropomyosin-related kinase B
tsDMARDs	targeted synthetic DMARDs
TTR	serum paraoxonase
VST	Victoria Stroop Test
WAIS	Wechsler Adult Intelligence Scale
